# Medical students' views on the value of trigger warnings in education: A qualitative study

**DOI:** 10.1111/medu.14803

**Published:** 2022-04-08

**Authors:** Helen A. Nolan, Lesley Roberts

**Affiliations:** ^1^ Warwick Medical School University of Warwick Coventry UK

## Abstract

**Background:**

Trigger warnings—advance notification of content so recipients may prepare for ensuing distress—feature in discussions in higher education. Students' expectations for warnings in some circumstances are recognised, and some educators and institutions have adopted use. Medical education necessitates engagement with potentially distressing topics. Little is known about medical students' expectations regarding warnings in education.

**Methods:**

All students from a 4‐year graduate‐entry UK medical degree programme were contacted via digital message outlining study details and were openly sampled. Qualitative methodology was chosen to explore participant expectations, experiences and meanings derived from experiences. Students participated in semi‐structured interviews exploring perspectives on functions, benefits and drawbacks of trigger warnings in classroom‐based medical education. We analysed interview transcripts using thematic analysis.

**Results:**

Thirteen semi‐structured, qualitative interviews were undertaken. Themes in the following areas were identified: (1) students' experiences influence understanding of trauma and trigger warnings, (2) warnings as mediators of learning experiences, (3) professional responsibilities in learning, (4) exposure to content, (5) professional ethos in medical education and (6) how to issue trigger warnings. Students recognised the term ‘trigger warning’, and that warnings are an accommodation for those affected by trauma. Students' conceptualisation of warnings was influenced by personal experiences and peer interactions both within and outside education. Students expressed both support and concerns about use of warnings and their ability to influence learning, assuming of responsibility and professional development.

**Discussion:**

Diverse student opinions regarding warnings were identified. Most students suggested that warnings be used prior to topics concerning recognised traumas. Incremental exposure to distressing content was recommended. Students should be supported in managing own vulnerabilities and needs, while also experiencing sufficient formative exposure to develop resilience. Greater understanding of trauma prevalence and impacts and underpinnings of warnings amongst students and educators are recommended to optimise education environments and professional development.

## Abstract 2

Medical students explain how trigger warnings mediate their learning experiences. While content exposure is essential, warnings can support understanding of trauma, empathy and professional development.

## 1 INTRODUCTION

Trigger warnings—prior notification allowing recipients to prepare for or avoid sensitive content and ensuing distress—are widely encountered in communications.^1^, ^2^ These advisories are considered to have originated online as an accommodation for survivors of sexual violence or other trauma and who may experience symptoms of post‐traumatic stress disorder (PTSD).^3^, ^4^ Although associated terminology may have changed, the practice and construct predate their online use.^3^ Their use has been widely adopted in relation to diverse settings and topics.^2^, ^5^ Discussion continues about their role in education, with evidence of some students expecting warnings in relation to distressing topics.^6^, ^7^ In some cases, educators and institutions have shared these sentiments, voicing support and citing rationale for adoption of warnings in practice or policy,^8^, ^9^ including desire to curate inclusive learning environments.^5^, ^10^, ^11^ Support has not been unanimous, with opposition to the construct, underpinning principles and use of warnings in education noted.^10^ Concerns expressed include promotion of avoidance,^12^ hypersensitisation of recipients^1^, ^13^, ^14^ and censorship effects.^12^, ^15^ Despite routine use and relevance to classroom settings, current literature regarding trigger warnings is largely derived from opinion pieces based on individual or few author perspectives and rigorous academic evaluations or empirical evidence regarding trigger warnings remain lacking.^16^

As an accommodation for affected individuals, trigger warnings and associated discussions have relevance to clinical education contexts where discussions of trauma, suffering and inequalities are integral and commonplace.^17^, ^18^ Graduating professionals need to regularly encounter and manage these subjects, while maintaining own well‐being. Medical students' perspectives in this area remain relatively unexplored. Inquiry may provide insights into students' experiences of distressing content, how best to prepare graduates for managing distressing experiences and whether warnings may have a role. Without student consultation, educators risk maintaining inconsistent approaches, outwith a framework for practice, culminating in suboptimal learning environments.

Sensitive or trauma‐related subjects in medical education may hold personal relevance for students.^17^, ^18^, ^19^ Experiences in medical education themselves have the potential to traumatise, irrespective of personal histories.^20^, ^21^ Although currently there is limited literature regarding secondary traumatisation in medical students,^20^ there is substantial evidence regarding depression and burnout amongst medical students,^22^, ^23^, ^24^ entities that are more prevalent amongst medical student than general and other student populations.^22^, ^23^, ^25^ These issues are further compounded by reported stigmatisation of medial students experiencing mental illness.^26^, ^27^ Incorporation of trigger warnings could promote accessibility by enabling reasonable accommodations for students with trauma histories or mental health difficulties^10^, ^11^ and signal that well‐being is valued in the organisation and profession, as previously described by medical educators.^28^

Diversity of medical student populations is increasing internationally, in response to measures to ensure representation of the served patient populations.^29^, ^30^ Significant increases in numbers of students admitted from educationally and socially disadvantaged backgrounds as well as groups from ethnic minorities and students experiencing disability^31^ are noted—all groups noted to experience higher incidence of adversity.^17^ Consideration of trauma‐informed approaches to distressing content, including use of warnings, appears increasingly justified and necessary in this context.^17^, ^32^

Given established impacts of emotion^33^ and PTSD^3^ on learning, efforts to identify evidence for efficacy of trigger warnings in general education literature have explored impacts on arousal and distress. One experimental study identified that trigger warnings' had ‘trivial’ effects on participants' ratings of negative material and distress symptoms,^14^ while acknowledging that warnings may have other effects not assessed by their study. Bellett et al.'s larger replication study overturned their original finding that TWs affect some domains of resilience, leading them to conclude that warnings are ‘inert’.^13^ They acknowledged that educators may use warnings for other reasons not explored in their study. Recruitment from non‐traumatised populations limits generalisability of these findings. Results of each of these studies^13^, ^14^ were also limited by reliance on participant self‐reporting of symptoms.

Evidence regarding trigger warnings in medical education is limited. Our previous semi‐structured interview study exploring the views and practice of medical educators identified that educators regularly employed warnings in classrooms settings.^28^ They cited various rationales beyond mitigating hyperarousal as well as a number of concerns relating to use of warnings. A single study of medical student perspectives, a subsection of a larger survey, exploring students' views suggested warnings may have a role in teaching about trauma but did not establish clear consensus regarding support for warnings.^34^ Survey methodology, however, prohibited depth of discussion. Despite clear relevance of trauma‐related content to medical student populations and experiences, and impetus to consider appropriateness of warnings in managing related impacts in education settings, medical student perspectives remain underexplored. This current interview study aimed to explore medical students' experiences and perspectives regarding the role of trigger warnings in classroom‐based medical education. Noting that experiences of traumatising content are personal to the individual,^17^ we wished to broadly explore students' perspectives and constructs of trigger warnings, including both within and outwith education experiences. As trigger warnings may have pedagogical function beyond preventing hyperarousal for individuals identifying as affected by trauma, we wished to explore perspectives of students identifying with varying personal experiences of adversity. We formulated the following research questions:

Do medical students perceive value in the use of trigger warnings? What are medical students' perspectives on the function, benefits and drawbacks of trigger warnings in classroom‐based medical education?

## 2 METHODS

In this study of medical students' perspectives and expectations regarding trigger warnings, we wished to explore social behaviours and experiences, meanings derived from experiences and factors underlying expectations of an educational phenomenon; thus, a qualitative methodology was used.^35^ Individual semi‐structured interviews facilitated deeper discussion of more complex questions allowing participants to share detailed accounts of experiences, interpretations and perspectives.^36^

The study was approved by University of Warwick Biomedical Research Ethics Committee.

### 2.1 Participants

Students on a 4‐year medical degree programme (*MBChB)* were recruited. Eligible participants needed to have completed at least 3 months of the programme, ensuring adequate experience of classroom‐based teaching, including lectures, case‐based learning and small group sessions. At the time of recruitment, the most junior students were nearing completion of first year, so were eligible to participate. Additionally, senior students could offer reflections on the appropriateness of early teaching as preparation for clinical practice. Unlike previous studies in this area, we did not limit participation to individuals who identified as not having experienced past trauma.^13^ Personal trauma history may influence perspectives and value participants assign to warnings. However, we also recognised that trigger warnings may be viewed as serving wider pedagogical function, including development of understanding and empathy towards trauma amongst non‐affected individuals, as suggested by previous studies^10^, ^28^, ^34^ *or*, conversely, impeding learning experiences; thus, all students on the 4‐year programme were openly sampled, capturing diverse perspectives and their evolution through programme progression.

Students were contacted via a digital message and provided with information outlining study details. The participant and recruitment information highlighted that responses would be anonymized and decisions regarding participation had no bearing on academic progression. We stated that we did not intend to directly explore distressing personal experiences, but that participants may refer to previous experiences in responses. Participants were informed of steps that would be taken if at any stage they needed support including discontinuing interviews and signposting to appropriate services. After addressing any questions, participants provided written consent to participate.

### 2.2 Context

Our programme is atypical in UK medical education as a *graduate‐entry* programme, compared with standard school‐leaver entry. Degree holders from any academic background are admitted. These criteria widen participation in medical education by traditionally underrepresented groups and in relation to student sociodemographic profile. Students are older, come from more varied backgrounds and have greater life experience. As an accelerated programme, ours is intensive and academically demanding; thus, student well‐being is emphasised in curricular development and delivery. Guidelines for practice in relation to warnings have been developed, based on a student feedback and disseminated amongst educators, and discipline teams have in some cases determined best approaches in their subject area. However, there is not currently an overarching institutional or departmental mandated policy on use of trigger warnings.

### 2.3 Data collection: Interviews

We developed interview questions aligned with our overarching research questions. As this study builds on a previous study of educators' views in this programme setting,^28^ these findings, in addition to existing literature and researcher discussion, influenced focus. Nonetheless, we wished to identify novel, unanticipated areas of priority for student participants. A semi‐structured interview approach with open‐ended questions was adopted to enable discussion of participants' experiences, with clarification and probing to qualify responses. Interview guide (Appendix S1) provides detail of questions.

As the study was conducted during the COVID‐19 pandemic and noting positive experiences of flexibility for participants, interviews were facilitated via Microsoft Teams videocall and recorded, unless participants chose an audio‐only call. Videos, where available, captured non‐verbal cues. Participants had the option of a socially distanced face‐to‐face interview if preferred; none selected this.

In the previous study of this subject with medical *educators*, the term *trigger warning* was not used initially, to accurately explore educators' use of warnings. As students may have experienced personally impactful content and, due to perceived power differential between students and faculty, it was ethically imperative to highlight intended discussion of trigger warnings and related circumstances during recruitment. Further, it was anticipated that students would readily recognise the term as it had been noted in student feedback. Students had an opportunity to clarify views on what constituted a trigger warning.

The interview guide was structured to initially explore participants' experiences of trigger warnings in day‐to‐day life and then in classroom‐based education. This initial, general approach sought to establish general familiarity with the subject in a less personalised way, before the possibly more contentious subject of warnings in education. Semi‐structured interviews allowed an approach that was often more fluid, enabling participants to freely discuss experiences and areas of greater priority to them. After initial interviews, a further question exploring previously unanticipated areas was added.

### 2.4 Data analysis

Thematic analysis was used to identify, analyse, organise, describe and report themes within the dataset. Here, a theme is a notable feature of the data relating to the research questions and adds meaning.^37^ Thematic analysis was chosen to assess participants' perspectives and identify similarities and differences in responses and unanticipated perspectives, creating a rich account of the data.^38^

HN transcribed verbatim audio recordings. We both read and reread transcripts for immersion and familiarisation. Analysis of initial interviews commenced as further interviews progressed. Any striking features and patterns noted in the data during collection, transcription and analysis were recorded and discussed. These were then incorporated in identifying preliminary codes. Further codes were identified, developing a full coding framework. HN iteratively coded all transcripts using Nvivo12. LR read all interview transcripts to triangulate and establish agreement. As new codes were identified, these were applied to previously coded transcripts. We reached consensus on suggested codes and how these were assigned to data. All data assigned a particular code were collated. The complete codebook was reviewed, searching for relationships and patterns, thereby identifying themes inductively. Diagrams were used to organise themes and subthemes. Proposed themes were compared with the dataset, confirming key findings had been reported. Titles of themes were then revised, ensuring appropriateness. Detailed notes were maintained throughout analysis, beginning in the transcription phase. Features and patterns in the dataset and codes and development and hierarchies of themes were documented in a reflexive diary, providing an audit trail.

### 2.5 Reflexivity, positionality

We both have leadership roles with oversight of student feedback and programme enhancement, including issues of accessibility and duty of care. LR has a senior educational leadership position and is known to students. HN has a quality leadership role and is directly known to fewer students. While involved in curriculum development, we are not substantively involved in programme delivery. Noting that roles may impact students' willingness to share experiences, HN conducted all interviews.

Regular researcher meetings occurred throughout the study, discussing reflexive notes and observations. Discrepancies in analysis, interpretation and reporting were considered, including regarding coding and theme titles. We maintained awareness of potential bias during analysis and actively sought evidence of contrary views, ensuring that our individual perspectives did not disproportionately influence interpretation or reporting.

## 3 RESULTS

We conducted 13 semi‐structured qualitative interviews with students (six males and seven females). Data collection occurred between June and October 2021. Average interview duration was 65 min (range 47–101 min). Participants from various backgrounds and from each of the four programme years participated (Table 1), sharing diverse perspectives and experiences and providing data relevant to the research questions and unanticipated areas. Noting the quality of dialogue, variety of participant perspectives and insights shared, we identified that we had appropriate data to address the research questions after completion of 13 interviews.^39^

**TABLE 1:** Overview of study participants

Participant number	Year of study	Gender
P1	Y2	M
P2	Y2	M
P3	Y3	M
P4	Y4	F
P5	Y3	F
P6	Y4	M
P7	Y1	M
P8	Y2	F
P9	Y3	F
P10	Y3	F
P11	Y2	M
P12	Y2	F
P13	Y2	F

In addressing research questions to explore student perceptions of value, function, benefits and drawbacks of trigger warnings, six thematic areas were identified, shown in Table 2. Quotes are presented by participant number and year of study.

**TABLE 2:** Summary of themes identified

Thematic area
How students experiences influence understanding of trauma and associated understanding of trigger warnings
Warnings as mediators of experiences in learning
Professional responsibilities in learning
Exposure to content Necessity of exposure to content Expectations of exposure
Professional ethos in medical education
How to issue trigger warnings

Support for warnings varied; individuals who identified personal need for warnings were exclusively supportive of use, whereas individuals who did not identify own need fell into two groups of supporters or opponents (fully or in part).

### 3.1 How students' experiences influence understanding of trauma and trigger warnings

Participants recognised trigger warnings as having a relationship to trauma and intended as an accommodation for individuals affected by trauma. Participants' familiarity with warnings arose both from mainstream media, including social media where they appeared to be employed ubiquitously, and experiences in education settings.

I'm pretty sure that social media companies … will screen things and put their own warning on saying ‘Sensitive topic … you might not want to see this’. (P5, year 3)

A small number had personal experience of trigger warnings from therapeutic contexts. For some participants, their understanding of warnings had changed from initially recognising them as entities used excessively (e.g., on social media), in innocuous circumstances and as incongruous with the aims of medical education, to being a reasonable consideration for individuals affected by trauma. Views had changed due to gaining insight into peers' perspectives.

Now I've spoken to people about it, I've got more … empathy with it. The triviality sometimes can water it down …. When someone explained how suicide, because of their own personal experience, really upsets them without a warning in lectures, you can understand the need for that … but I find labelling it as ‘trigger warning’ trivializes it, like it's less serious. (P9, year 3)

Participants speculated about the prevalence of sensitive or active trauma issues in their student population, some noting that programme choice implied readiness for exposure to illness and suffering.

I'd be … surprised if somebody had a … breakdown or cried every time they heard of a sick patient, …. I'm presuming most people on the course would be able to handle it. (P13, year 2)

Participants considered whether warnings were necessary or appropriate in medical education, highlighting that the course necessitated exposure to illness and trauma in preparation for clinical careers. Some indicated that this overarching prerequisite necessitated absence of such warnings and this, with appropriate well‐being support, enabled development of professional self‐care.

We're adults, we have to take ownership over our own well‐being. And it's fantastic, the support we are offered … I do not think you need to blanket warn everyone on a regular basis. (P2, year 2)

Others, however, recognised that that warnings anticipated rare or exceptional responses and catered to a minority with specific experiences.

To me, they may seem a bit useless at times, but I can imagine, for example, if you are recovering from an eating disorder, and someone starts talking about behaviours, that might be quite triggering to you. (P10, year 3)

There was also recognition that beyond specific topic areas, trauma was not uncommon and that this may be associated with increasing self‐awareness and emotion recognition.

My generation are known as the snowflake generation. And maybe are we are more emotionally sensitive … I do not think that's a bad thing. (P10, year 3)

Some participants mentioned that warnings had attracted peer debate. They noted that while proponents advocated for use of warnings, not all students' views were represented, and important perspectives were omitted:

That approach where you are giving content warnings, but you are not making a thing of it appeases both groups … They're just the loudest, they are not the most common, the most common person is happy with whatever they are given. (P12, year 2)

### 3.2 Warnings as mediators of experiences in learning

Participants expressed concerns about circumstances where warnings may be requested to facilitate avoidance:

I do not think that should be with the intention to avoid it, I think that should be with the intention to prepare themselves as part of their healing process and learning how to manage the trigger in future. I think that if they stated the intention [is] so that they can avoid that content … I would see that as a bit of a red flag. (P1, year 2)

Those opposed to warnings reported that they disrupted flow of delivery and suggested warnings had potential to be distracting and cause possible nocebo effects for others:

It really disrupts the flow … people start thinking; “Oh, do I need to be upset about this?” rather than thinking about the case. (P13, year 2)

Other participants discussed that scenarios in education could have ‘triggering’ effects:

An emotional response, something like shock, fear, anxiety … which can be quite extreme, if you suffer from certain mental health disorders or you struggled with particular traumas in your life. (P11, year 2)

Participants explained that this impeded learning:

A strong, detrimental emotional response in you, whether that's because it's generally upsetting, or it resonates with something you have experienced before … then it becomes very difficult to stay with the thread of what's going on … because you are off down in your own rabbit hole. (P8, year 2)

Warnings could allow recipients to prepare themselves by regulating emotional responses, and they informed recipients, granting control and choice regarding the circumstances of engagement with distressing content. Some consensus was noted regarding circumstances where warnings were appropriate. Frequent suggestions included discussions of sexual violence, domestic violence, racism and racialised violence, suicide, eating disorders, ethical issues, and pregnancy loss.

Participants proposed that warnings could also help other unaffected students to develop understanding of issues with personal impact and support development of empathy with others' lived experiences.

If you start highlighting that this could be sensitive for people, you are showing that these are important things that cause trauma … I think it benefits people who do not know that things are upsetting, it might actually give them insight to their feelings and then signposting them to support services. You're potentially helping people gain insight. (P9, year 3)

Here, participants suggested that warnings afforded benefits to unaffected students by enhancing awareness and empathy for others:

It's also partly educational to others who may never feel triggered by that particular thing, it educates people that actually there are people who might find this triggering … It has an educational purpose, which is secondary, but important. (P11, year 2)

### 3.3 Responsibilities in learning

Participants frequently discussed educators' and students' professional responsibilities in the educational process, which included respective responsibilities to provide and engage with content that could be distressing. There were a range of views in relation to expectations of individuals and where the balance of responsibility lay within that relationship.

A small number of participants appeared to view the duty of care and accountability for student well‐being to be predominately the remit of the medical school, with students being recipients of education and care.

As lecturers … you are here to look after us … make sure we are okay. So, providing support or where to get that is part of your job, I'd say. (P5, year 3)

Others highlighted that, owing to the risk of educators receiving complaints, educators may use warnings for defensive or self‐protective reasons and that these drivers may conflict with educators' own ethos.

I think it's a way of somebody kind of shifting liability off themselves. (P12, year 2)

Others described education and professional development as a partnership between educators and students. They discussed students' responsibilities within that model, which included having awareness of and managing one's own vulnerabilities. Students have a duty to disclose personal issues that may impact or be impacted by their education, so that these could be proactively managed, ensuring an effective learning experience. For some participants, this was a preferable approach that supported development of professional coping skills, without educators needing to speculate about distressing issues or embed avoidance strategies through implementation of widespread trigger warnings.

It is that person's responsibility to disclose that to the medical school … to seek help before you encounter that. (P2, year 2)

General acceptance by students of their own responsibilities and self‐directedness was necessary, as a significant proportion of learning in medical education is self‐determined by learners:

I think it's what you expose yourself to … in medical school, especially in the clinical years, your learning is very self‐directed, you have to get your own opportunities. (P10, year 3)

Some participants felt that warnings divested students of this responsibility and risked underestimating their coping ability, leading to infantilisation, curtailing exposure and developmental opportunities. This was incongruent with their identity as adult learners.

Being a doctor is not easy, I do not want to necessarily be mollycoddled my entire way through medical school, so that then when I jump into the real world, I actually have not got coping strategies. (P2, year 2)

Here, omission of warnings did not equate to disregarding student welfare.

I do not think that being pre‐warned is the best way to learn how to cope, it's not not caring about someone's feelings… We learn in medicine you sometimes actually cause a bit of pain to help someone. And giving trigger warnings, are we preventing the student from … coming to terms with whatever troublesome thing they are facing…? (P3, year 3)

There was value in students learning to tolerate negative emotional reactions that would be relevant to experiences such as professional failure or poor patient outcomes.

Others similarly discussed the need for students' self‐awareness and self‐management of vulnerabilities. However, they did not view warnings as being antagonistic to students assuming responsibility and developing self‐awareness. Where warnings were provided as overview statements of content, these fulfilled the function of informing recipients, prompting them to reflect and determine their readiness or need for support. A gradual approach to withdrawal of warnings could scaffold students' self‐awareness and self‐reliance, allowing development and assumption of responsibility over time and enabling self‐identification and action planning regarding sensitivities.

There's a certain element of autonomy and self‐awareness that goes into triggering content … There's an onus on the individual, especially if you are unexpectedly triggered, to seek help, or think about that, and then work out how you are going to be in future with it. It's not up to the [educator] to have to pre‐empt all of that. (P8, year 2)

For some, a warning was only useful if it prompted the affected recipient to take appropriate action:

I think it's only useful if it's paired with a strategy to engage voluntarily… If it's just on its own, people can get by completely avoiding it, and when they are confronted in real life, it's going to be a hundred times rawer. (P7, year 1)

Framing self‐awareness and self‐care as professional attributes could encourage student ownership, engagement and identity development.

Seeing it as part of keeping yourself well and being a professional … I think professionalism is seen as a dirty word … like it's something to be caught out by. But actually, it's keeping yourself skilled and as healthy as possible, to do a good job … and you should be proud of that rather than worrying. (P9, year 3)

Additional effective approaches to addressing distressing content in partnership included educators' duty to debrief or explain the educational and contextual relevance of material with students and for students to be curious and willing to explore that.

I think there's an onus on [educator] to be approachable … we hope … that debriefing process is a two‐way street …. so the student can equally hear why that content is necessary and important to cover. (P8, year 2)

### 3.4 Exposure

#### 3.4.1 Necessity of exposure

Central to discussions of distressing content and warnings in education were considerations of exposure. Participants acknowledged that exposure and engagement were essential to ensure professional preparedness. Exposure to distressing content was inevitable in unpredictable clinical learning environments.

You have to be prepared for those things. In real life you do not get trigger warnings… You do not get a note over patients saying “this patient's going to collapse now ‐ watch out”. (P13, year 2)

For some participants, exposure provided during medical education was a valuable opportunity and should not be attenuated. They identified exposures to emotionally challenging content as useful developmental opportunities. Through exposure and subsequent processing and reflection, they developed coping mechanisms in a self‐directed manner. Such experiences were not harmful but precursors to requisite professional resilience.

I prefer to have that experience and see how I've coped, how I've adapted. I do not want to always feel patronized if I'm being told “here's a trigger warning” … For me it's more empowering to know I've faced this problem, I've dealt with it, and I'm stronger for it. (P3, year 3)

Other participants, who too had acknowledged that exposure to distressing content was not only unavoidable but important, discussed that development of resilience starts from different baselines in individuals and that it was appropriate to take deliberate measures in managing such content. These may include ascertaining the educational role of content and providing incremental exposure throughout the programme. Avoidance was not the intention, but exploration of more impactful, potentially distressing content, could be deferred until students had amassed coping skills and clinical experience.

We're not qualified, we are preparing. Part of that is smaller steps and accommodations … you would not expect an apprentice bricklayer to build a mansion in one day—sometimes you may miss a brick, and need help. (P12, year 2)

Warnings were another appropriate measure to allow students to be informed, to prepare themselves and to have control regarding circumstances of exposure. One participant, who highlighted that incremental exposure was a reasonable expectation, used an analogy to drug dosing in discussing this progressive approach;

I expect to get to the point, by the end of my degree, where I am more comfortable with those things than at the start. It's about helping create resilience to those things, and almost like dosing slowly over time, being able to reduce my reaction over time. And being able to do that at a pace that I want, the way I choose to engage with it. (P11, year 2)

By enabling students to have agency and control their exposures, via warnings, students were allowed to be self‐directing.

If they want to experience it to become accustomed, they can choose to do that themselves, and … not have that forced upon them … it could actually harm their mental health. If a person has a trigger warning and thinks “Actually, I might sit in for this”, that's their choice. (P5, year 3)

#### 3.4.2 Expectations of exposure

Although participants accepted, and often welcomed, exposure to challenging content, there was evidence of differential expectations and tolerance in different settings. Participants discussed how mindsets in classrooms and clinical environments differed. Distressing content was more likely to be encountered, and therefore anticipated, in clinical settings than classrooms.

It's about your headspace. When I drive into uni, I'm not in hospital mode, I'm not ready to see those things. When I go to the hospital, I'm ready to see them. I'm prepared. (P10, year 3)

You're expecting that sort of thing when you are in A&E, you … know that might come in, you can prepare yourself mentally for it. You're not really expecting a trigger warning there. Whereas … in a lecture … some sort of warning would be nice. You're not in the headspace to have to be accepting these things. (P5, year 3)

The classroom was primarily associated with learning and students identified their personal learning as being of greatest priority. It was therefore expected to be a safe, supportive environment. Where exposure to distressing content featured, warnings could allow students to prepare themselves.

Within an education setting … you are in an environment which is supposed to be nurturing and supporting you as a student. When you are in placement … it's a professional capacity … When you are sitting in a classroom, you are expecting to … be able to think … reflect … ask questions. In a clinical setting you are expecting to see people who are unwell. (P8, year 2)

### 3.5 Professional ethos in medical education

Participants considered how warnings functioned within the professional culture and expectations of clinical education and training. Some participants described reticence to unreservedly discuss current or anticipated mental health difficulties at admission. This was particularly true of participants with a personal need for warnings. Factors cited included fear of not being admitted to the programme, concern about being withdrawn and displeasure at being negatively evaluated.

I did not delve into detail on my application because I was very worried … people would reject me purely on that. A lot of people fear that if you show that you have some anxiety or whatever, they'll kick you off the course … that's a big fear…. (P4, year 4)

Participants feared stigmatisation from both peers and educators due to vulnerabilities and identified that warnings were necessary in preparing to engage with curricular content.

I did not want people to think I was a weak medic or not fit for the job. (P4, year 4)

Educators could consider various inter‐related factors in their approach and treatment of educational content and environment.

I think people should be more mindful of what can cause triggers …. having an ethos of support, and making sure they are cognizant of these issues, and the impact … on students. (P6, year 4)

Participants discussed the role of warnings in signalling compassion and empathy for affected individuals and that their needs were accepted in the education setting. Further steps could be taken to overturn traditional, unhealthy expectations of medical professionals and to validate students' acknowledgement of their own limitations.

It's important to consider that just because someone was triggered one day, it does not mean they are going to be triggered another …. Everyone holds these images of doctors, we still have this image of them being …. impenetrable …. I think people want to live up to that. (P10, year 3)

The manner in which trauma‐related content was presented and managed impacted on student's sense of safety and belonging in medical education. One participant discussed negative personal impacts of omission of warnings. Current approaches appeared to perpetuate traditional stereotypes in medicine.

I feel like there should have been a level of pre‐warning … an acknowledgement that what you might experience could be triggering. I feel like the medical school …. does not actually think that we might have even had these experiences ourselves … because there's only a certain type of person that does. (P5, year 3)

For warnings to fulfil a supportive function, they should be informative and pastoral in intent, and not be coupled with professionalism requirements:

I do not think talking about [professional] preparedness should be ever linked with trigger warnings …. … It's really important when you are talking about a trigger, that you are only focusing on that, not to say, “by the way, you need to heal a little bit faster” that person does not need that pressure …. (P12, year 2)

Participants expressed desire for greater acknowledgement of challenges experienced during students' journey to becoming a doctor and compassionate role modelling by the organisation.

I think our lecturers should be compassionate … and know that we have experiences that we might not want to share … they are training us to be doctors, hoping that we are compassionate, so I'd hope that they'd be the same. (P5, year 3)

Greater integration of trauma‐related content in the curriculum would promote understanding of its prevalence and impacts, reducing stigma;

It needs to be talked about more at the med school ‐ trauma, and mental health … make it more of a priority … bring it into [cases] about patients being triggered … (P12, year 2)

### 3.6 How to provide warnings

Participants discussed the principle of providing advance notification and how warnings were given. Some students who expressed limited support for warnings and underlying principles conceded that use may be appropriate in limited circumstances related to recognised traumas. Wide variation was noted regarding the most appropriate way to do this and the primary rationale. Several participants expressed disapproval for the term due to connotations associated with *trigger warnings*: the expanded use to encompass content of widely varying severity and as a tool that could permit avoidance or stifle debate. Others, noting that warnings presupposed or could precipitate harmful responses, proposed alternative terminology such as *content statements*, providing objective information. This measure, coupled with signposts to supports, delegated responsibility to students as partners in the education process to consider own needs.

Where someone just says, “we are going to be discussing this today” … so it does not really come as much of a surprise. “If you find this content upsetting then please feel free to step out of the room and re‐join when you feel comfortable”. I think that's a bit more sensitive to people … giving them information and ground rules, as opposed to just announcing, “Trigger warning!”, because it's … a thing that needs to be done. (P1, year 2)

Others expressed expectation for directive warnings in some circumstances, demonstrating educators' compassionate acknowledgment regarding the impact of content on students. Some participants did not feel that regular forewarnings were a justifiable expectation, preferring to be informed during induction regarding the breath and nature of content that would be encountered and how to access support, if required.

## 4 DISCUSSION

This study sought to explore medical students' experiences, perspectives and priorities regarding use and value of trigger warnings in classroom‐based education. In contrast to our previous study with educators, all participants readily recognised the term from contexts including media, therapeutic and educational settings. In keeping with previous findings, arguments both in favour of and against use of warnings were presented.^3^, ^28^, ^34^, ^40^ Opponents expressed concerns that use of warnings made inferences regarding students' limited coping abilities. Others cited drawbacks similar to those identified by educators,^28^ including disrupting flow of discussion and hypersensitising recipients. Warnings could be primarily used as defence against anticipated complaints. Although warnings were a concession required by a minority, they were imposed on all students. Some suggested that enrolment to medical education implied readiness to encounter routine content and that the need for warnings was overstated, implying trauma was not a concern for this population. More participants recognised a role for warnings, due to personal need or as reasonable concessions for others. Trauma‐related content was recognised as causing adverse emotional reactions, impeding learning, which could be ameliorated by advance warnings. The role of warnings appeared to have been debated amongst some students, leading to occasional discordance and polarisation in views.

A key aspect of contention surrounding warnings has been whether they promote avoidance of distressing content or facilitate engagement,^5^ a variance also noted in this study. Participants offered contrasting perspectives regarding how warnings moderated engagement, supporting or destabilising learning. Some participants desired authentic experiences and unmoderated challenge, promoting personal stretch and development, with such challenge being requisite to transformative learning^41^ and development of professional resilience and readiness for clinical practice. Warnings were seen by some as prohibitive to achieving these outcomes. Attenuation of anticipated emotional responses was not considered necessary as such responses were not harmful but were important precursors to resilience. Others expressed similar ultimate ambitions for professional readiness but identified that warnings allowed students to prepare themselves by establishing an appropriate mindset to deal with distressing content, a habit that was already practiced in relation to clinical environments, where distressing content was likely to present. Warnings had a role in enabling safe, self‐directed engagement and self‐awareness.

Participants discussed responsibility for learning and managing individual sensitivities in education settings. Students, as developing professionals, recognised the realities of medical education and the requirement for self‐awareness in managing their own needs.^42^ Duty to acknowledge and take responsibility for this was noted. Elsewhere, evidence was noted of students' identifying these responsibilities as lying with educators, suggesting an external locus of control that may be at odds with readiness for self‐directed learning.^43^, ^44^ Warnings could thereby contribute to disempowerment. Others highlighted that educators typically controlled session content, further contributing to the power differential between educator and student and placing a responsibility on educators to consider recipients' needs.^5^ In undertaking a journey to professionalisation, students commenced in a novice role. Models of self‐directed learning highlight that learners' progress through different stages of self‐directedness and that self‐directedness traits can be acquired, nurtured and developed.^45^ Professional preparedness could be acquired through incremental, scaffolded approaches, where warnings accommodated individual needs. It has previously been positioned that future professional responsibilities should not be prioritised over students' own current needs^28^ and self‐care should be enshrined in professionalisation.^46^, ^47^

Participants who identified a personal need for warnings desired compassion and acknowledgement from educators and that classrooms be preserved as nurturing environments, as indicated by differential expectations in classrooms and clinical environments. Where practice conveyed educators' consideration towards students, this demonstrated acceptance of students' needs and circumstances, a suggestion also proposed by educators.^28^ Professional identities are influenced by the culture of learning environments and processes of socialisation.^48^, ^49^, ^50^ Caring attributes, expected in future professionals, should be upheld and role‐modelled in these contexts.^49^ Participants explained how educators' treatment of trauma‐related content was indicative of organisational attitudes and professional norms. These experiences impacted sense of belonging and identity and relationships with peers and the organisation, factors noted as inherent to professional identity formation.^49^ These hidden curriculum experiences echoed those of students from previously underrepresented backgrounds.^51^ Intersections of the experiences of traditionally underrepresented students, including minority group experiences, power hierarchies and social inequalities, with trauma provide further impetus for consideration of trauma‐informed approaches,^17^ including content warnings, for increasingly diverse student cohorts.

Participants shared perspectives regarding personal growth arising from exposure to challenge and stress. An optimal degree of stress—‘eustress’—can be motivational and performance enhancing.^52^, ^53^ Trauma, a related but distinct experience, characterised as more severe and with persisting adverse effects,^54^ and positioned further along this stress spectrum, was also discussed. Stress and trauma are opportune areas to explore in medical education, an experience widely acknowledged as intensive and having potential to harm practitioner well‐being.^20^, ^21^ Trauma‐informed medical education advocates for integration of trauma‐informed approaches in curricular development, delivery and learning environments.^17^, ^19^ This includes teaching about science underpinning trauma and its effects, acknowledgement of potential impacts of trauma‐related content on students, and accommodation for this through use of content overviews and advance warnings, thereby promoting understanding amongst students and educators,^17^ a recommendation shared by some participants. Greater evidence‐based understanding of stress and trauma, as both affordances and hazards, may be achieved through use of warnings, promoting empathy and avoiding marginalisation of either supporters or opponents.

Considerable variation in views was noted regarding the primary intended purpose of warnings and the best way to provide these. Many participants were broadly supportive of the principle of providing advance, objective information regarding content and that a small subset of more impactful topics be managed using more directive advisories. Participants acknowledged concerns about warnings being delivered in an alarmist fashion, used inconsistently or solely for defensive reasons. As with educators, there was limited support for the term *trigger warning*,^28^ noting that this pre‐empted a negative response and may be considered a concession for a supposed ‘snowflake generation’. Solutions offered included objective content statements that served required functions of granting choice to recipients.

Warnings remain a contested construct with dissonance noted regarding their primary function and mechanism of action,^5^, ^10^ observations also noted in the current study. Medical educators have suggested that warnings have broad functions, including empowerment, enhancing student self‐awareness and supporting safe engagement,^28^ views echoed by participants here. A warning could allow recipients to regulate their response. Emotion regulation (ER)—the monitoring, evaluation and modification of emotional responses^55^—impacts academic outcomes when employed by students.^56^ Regulation by cognitive or arousal reappraisal, which aims to change the type of stress response, encourages individuals to reconceptualize stress as a coping tool.^57^ This technique has been explored in both therapeutic contexts,^58^ resulting in decreased PTSD symptom severity,^59^ and academic contexts,^41^ showing effectiveness in improving student outcomes.^60^ Compared with other regulation strategies, specifically suppression, cognitive reappraisal was associated with lesser symptom severity in PTSD^58^ and lower levels of academic burnout.^56^ No evidence was identified here, or in our previous study, to suggest warnings were requested or used primarily to enable such avoidance but instead to facilitate engagement. Although these approaches do not replace appropriate therapy for students affected by trauma, they may provide insights into how warnings could be reoriented and delivered more effectively, and as an adjunct to appropriate treatment, to support engagement.

Warnings are not a panacea for managing emotional responses and experiences in diverse medical student populations but may be one intervention that merits consideration. Other suggestions identified in this study included framing dual responsibilities within the education partnership. Educators had a duty to consider students and provide clear, advance content information. Students had a reciprocal responsibility to review this information, being mindful of own vulnerabilities, and proactively seek support if required. Objective content statements should be used consistently, with more directive measures for more severe content. Post‐exposure support and empathic educator responses should remain accessible to students.

## 5 LIMITATIONS

The study was conducted at a single UK *graduate‐entry* medical school that may limit generalisability of findings to school‐leaver entry populations. However, typical, younger school‐leaver entrants may not have had sufficient experience to develop professional maturity and resilience, meaning issues of vicarious traumatisation are pertinent. Furthermore, diversity in graduate‐entry populations, with students from traditionally underrepresented backgrounds, enhances representativeness, meaning that a diverse population was sampled. Experiences of adversity and trauma may be more likely in this population, allowing these participants to provide richer, more nuanced insights.

Unlike previous studies regarding trigger warnings, we did not limit participation to individuals identifying as having no past history of trauma.^13^ Arising from ethical considerations, we did not explicitly inquire about individual trauma histories but facilitated discussion of such experiences when volunteered by participants. Absence of trauma history categorisation of participants may be considered a limitation. However, noting suggested broader pedagogical functions or drawbacks,^10^, ^28^ and pervasiveness of trauma in medical education and practice,^17^, ^21^ we recognised the relevance of trigger warnings to all students.

Students with more severe traumatic histories may have been reluctant to participate, despite assurances in recruitment information regarding confidentiality and that past traumatic experiences would not be explicitly explored by the researcher. Further, both researchers have education leadership roles at the study setting and were aware these roles could lead to student reticence to participate or discuss experiences. However, we captured a variety of participant perspectives in relation to the research questions, including reflections on both trauma and resilience required of clinicians. Participants also shared experiences of trigger warnings in both therapeutic and educational contexts. These two points provided assurance of an adequate sample. Expanding the study to additional settings and increasing the sample size would capture further perspectives and enhance generalisability of findings.

## 6 IMPLICATIONS FOR MEDICAL EDUCATION

Some consensus has been established amongst participants regarding principles of providing balanced advance information to students about content and the importance of self‐directed engagement. Trigger warnings have been encountered by participants both in and outwith education environments. In the absence of consultation and agreed expectations of both students and educators, there is potential for dissonance and polarisation in students' views and experiences, with adverse impacts on peer support, and safety and effectiveness of learning environments.^3^, ^61^

Trigger warnings remain an ill‐defined construct whose use brings both opportunities and drawbacks. Suggestions for more acceptable terminology are noted, and there appears to be an opportunity to reorient approaches, harnessing pedagogical, pastoral and developmental benefits. Broader understanding of the intentions and limitations of warnings, in the context of evidence‐based understanding of stress and trauma, is recommended, as part of implementing trauma‐informed approaches. Managing distressing content can be undertaken within a partnership, enabling students to engage with content information, thereby developing self‐awareness and coping skills, and ensuring access to appropriate learning opportunities. Evidence‐based approaches such as arousal reappraisal techniques may merit further exploration. Educational environments that are nurturing, accepting of students' needs and appropriately challenging are required. Educators should continue to demonstrate empathy and consideration of the experiences and preferences of all students, including those with trauma histories.

## CONFLICTS OF INTEREST

We declare that we have no competing interests.

## AUTHOR CONTRIBUTIONS

HN conceived and developed the idea for the project, interviewed participants and analysed data and identified themes. HN drafted the early versions of the manuscript and made subsequent critical revisions for important intellectual content. LR developed the idea for the project, analysed data and identified themes. LR reviewed the early versions of the manuscript and made substantial contributions to the content and direction of the manuscript. Both authors approve the final version and agree to be accountable for all aspects of the work including questions related to the accuracy or integrity of the work.

## ETHICS STATEMENT

This study was reviewed and approved by University of Warwick Biomedical Sciences Research Ethics Committee (BSREC): BSREC reference number BSREC 114/20–21.

## Supporting information


**Appendix S1.** Supporting Information
