# Supplementary material for: Medical students' views on the value of trigger warnings in education: A qualitative study
Source: Med Educ. 2022 Apr 8;56(8):834–46. doi: 10.1111/medu.14803 (PMC9544229; doi:10.1111/medu.14803)
Supplement: Supplementary file 1 — Appendix S1. Supporting Information [file MEDU-56-834-s001.docx]

**Appendix 1; Interview guide for semi-structured qualitative interviews**:

1. Welcome, introductions. Before we start do you have any questions?
2. Before returning to study graduate entry medicine what was your previous role or occupation?

**Students’ familiarity with trigger warnings and views on their general use**

2. When you hear the term trigger warning, what does that make you think of?

1. Where have you encountered this term or concept previously?

It is anticipated that participants will discuss general use of trigger warnings e.g., in news reports, online and social media here.

For participants not familiar with this term it will be described by the interviewer and examples will be given participants understanding and recognition of the term and concept.

1. Watch do you see as the purpose of trigger warnings?
2. Do you think trigger warnings are useful? If so, in what circumstances?
3. Do you think trigger warnings are problematic? If so, why?

**Students’ experience with warnings in the education setting and views on their use here.**

1. Have you encountered any warnings being used in teaching or training situations? Can you describe this situation? This may include live classroom-based teaching, group discussions on written study materials or recorded learning resources or any other examples that the participant mentions.
2. Do you consider the warning you have mentioned here as being a trigger warning?
3. What purpose do you see the trigger warning serving in that situation/ those situations?
4. Do you think a warning was helpful in that situation? If so, why?
5. Do you think a warning was problematic in that situation? If so, why?
6. Do you think we should use warnings more often in medical education teaching or training situations? If so, when and why? Would there be any drawbacks to this approach?
7. Do you think we should use warnings less often in medical education teaching or training situations? If so, why? Would there be any drawbacks to this approach?

**Further question added after initial interviews**

1. In light of the challenges/ drawbacks and potential benefits discussed, how can/ should medical education implement more consistent use of warnings, in light of these limitations?
